# Promoting and Protecting Human Milk and Breastfeeding in a COVID-19 World

**DOI:** 10.3389/fped.2020.633700

**Published:** 2021-02-03

**Authors:** Diane L. Spatz, Riccardo Davanzo, Janis A. Müller, Rebecca Powell, Virginie Rigourd, Ann Yates, Donna T. Geddes, Johannes B. van Goudoever, Lars Bode

**Affiliations:** ^1^University of Pennsylvania School of Nursing, Children's Hospital of Philadelphia, Philadelphia, PA, United States; ^2^Institute for Maternal and Child Health Institute, Istituto di Ricovero e Cura a Carattere Scientifico (IRCCS) Burlo Garofolo, Trieste, Italy; ^3^Department of Virology, Institute of Molecular Virology, Ulm University Medical Center, Ulm, Germany; ^4^Division of Infectious Diseases, Department of Medicine, Mount Sinai Hospital, New York, NY, United States; ^5^Regional Human Milk Bank (Ile de France), Hôpital Necker Enfants Malade, Paris, France; ^6^International Confederation of Midwives, The Hague, Netherlands; ^7^School of Molecular Sciences, The University of Western Australia, Crawley, WA, Australia; ^8^Emma Children's Hospital, Amsterdam University Medical Centers (Amsterdam UMC), Amsterdam, Netherlands; ^9^Department of Pediatrics and Larsson-Rosenquist Foundation Mother-Milk-Infant Center of Research Excellence (MOMI CORE), University of California, San Diego, La Jolla, CA, United States

**Keywords:** breastfeeding, human milk, COVID, evidence based, paradigm

## Abstract

The global COVID-19 pandemic has put enormous stress on healthcare systems and hospital staffing. However, through all this, families will continue to become pregnant, give birth, and breastfeed. Unfortunately, care of the childbearing family has been de-prioritized during the pandemic. Additionally, many healthcare practices during the pandemic have not been positive for the childbearing family or breastfeeding. Despite recommendations from the World Health Organization to promote early, direct breastfeeding and skin to skin contact, these and other recommendations are not being followed in the clinical setting. For example, some mothers have been forced to go through labor and birth alone in some institutions whilst some hospitals have limited or no parental visitation to infants in the NICU. Furthermore, hospitals are discharging mothers and their newborns early, limiting the amount of time that families receive expert lactation care, education, and technical assistance. In addition, some hospitals have furloughed staff or transferred them to COVID-19 wards, further negatively impacting direct care for families and their newborns. We are concerned that these massive changes in the care of childbearing families will be permanently adopted. Instead, we must use the pandemic to underscore the importance of human milk and breastfeeding as lifesaving medical interventions. We challenge healthcare professionals to change the current prenatal and post-birth practice paradigms to protect lactation physiology and to ensure that all families in need receive equal access to evidence-based lactation education, care and technical assistance.

## Introduction

Within months severe acute respiratory syndrome coronavirus 2 (SARS-CoV-2) spread around the world, causing severe cases of coronavirus disease 2019 (COVID-19) and affecting health, economy, and society as a whole. The prioritization during the pandemic has been the care of the elderly and severely affected adults. However, the pandemic has also raised concerns for pregnant women and breastfeeding mothers. Newborns have been separated from their mothers and some health professionals and parents feared that human milk could transmit the virus from mother to child. This is despite the fact that from the beginning of the pandemic, the World Health Organization has endorsed early continuous skin to skin contact (SSC) and direct breastfeeding (1).

Rapidly changing recommendations on perinatal care induce stress and anxiety in pregnant women and in those who have just delivered a child (2). Families received conflicting information about allowing partners to be with them for childbirth, about whether or not direct breastfeeding and skin to skin contact were allowable, and conflicting advice about follow-up care. Consequently, both breastfeeding initiation and duration rates are at much risk for declining globally. While the authors acknowledge that some individual institutions have followed recommendations provided by the WHO, this has not occurred systemically in any country and in fact some countries such as China actively discouraged breastfeeding. While some individual hospitals may be providing expert evidence based care following WHO guidelines, this is not the cultural norm during this pandemic.

This is of particular concern since breastfeeding is a lifesaving medical intervention for children, decreasing both mortality and morbidity (3). The provision of human milk optimizes a child's short- and long-term brain development, intelligence and developmental outcomes (3). Children who receive human milk are healthier and more productive members of society (3). Breastfeeding further improves the mothers' health by decreasing her risk of breast cancer, heart disease, post-partum depression, obesity, and diabetes (3–7). The potential decrease in breastfeeding rates also negatively impacts our society overall with less healthy families, increased cost burden to parents for the purchase of infant formula, and increased cost burden to our society in terms of suboptimal health and development outcomes, health care expenditure, and impact on the environment (3).

Birthing practices, in particular, have changed markedly during the pandemic; for example, one facility in Italy contracted its maternal-infant ward non-COVID-19 inpatient activities by 30% (8). In a pragmatic, crowd sourced survey from the Infant Microbiome COVID-19 Project involving 328 healthcare professionals in 47 countries worldwide (9), only 6.5% of respondents reported no change in their institution's birthing practice. Half of all respondents (156/328) reported that mothers had to be alone upon entering the hospital. Most respondents (82%, or 259/328) also stated that only one support person was allowed for the childbearing woman. Additionally, 46.1% of respondents reported that only one parent was allowed to visit a child admitted to the Neonatal Intensive Care Unit (NICU). Respondents also reported other changes such as higher rates of induction (23.2%) and early admission for labor (7.7%). Interestingly, the requirement of the birthing parent to wear a facemask was only reported in 30% of respondents.

In a survey of mothers in five European countries, the proportion who reported that COVID-19 had had a negative impact on their breastfeeding or pumping behaviors ranged from 8% in Germany to 21% in France (10). Interviews conducted with first time COVID-19 negative mothers in the United States also found that birth and breastfeeding experiences were disrupted. Mothers reported that recommendations were changing daily and that they experienced significant stress and anxiety giving birth during the pandemic (2).

## SARS-CoV-2 and Human Milk

Some viruses such as human immunodeficiency virus (HIV) and human cytomegalovirus (HCMV) are shed into human milk and may be transmitted to the neonate, whereas for other viruses this has not been observed, e.g., lymphocytic choriomeningitis virus, measles (11, 12). When a new virus emerges, it is important to determine if the virus can shed into human milk and be transmitted to the breastfed infant. In the case of coronaviruses, the likelihood of vertical transmission is low, with no documented cases occurring in the past with either SARS or MERS (13). For SARS-CoV-2 the detection of viral RNA in human milk from infected women appears to be uncommon and transmission via human milk has not been recorded. Furthermore, the detection of viral RNA has only been found sporadically in samples of human milk from women infected with SARS-CoV-2 (14–20); with no milk samples showing active, replication-competent virus (18), as such, in no study has infectious virus been isolated from milk.

When active SARS-CoV-2 is added into human milk in the laboratory it remains infectious *in vitro*, but can be completely inactivated by Holder pasteurization (18, 21, 22). Whilst these studies have been performed with milk from uninfected mothers, recent data from infected mothers revealed that milk samples exhibited a robust SARS-CoV-2 spike-specific secretory immunoglobulin (sIgA) activity that might reduce viral infectivity (23, 24). As such, the ever accumulating evidence overwhelmingly indicates that human milk itself is safe and not a vector for viral transmission.

With respect to the anti-viral components in human milk it is important to determine if these may benefit the human milk fed infant and potentially help to either fight the disease or protect the infant from acquiring the disease in the first instance. Powell et al. are conducting a prospective study of over 800 women who have recovered from COVID-19 with each participant providing milk samples every 3–4 weeks, allowing for the longitudinal analysis of the immune response to SARS-CoV-2 in human milk. In a preliminary analysis it was found that 95% of milk samples contained anti SARS-CoV-2 sIgA (23). These data have been supported by Pace et al. (24) and van Keulen et al. (25), with all studies showing a robust sIgA response to SARS-CoV-2 and the ability of these sIgA to neutralize a SARS-CoV-2 clinical isolate strain.

In addition to SARS-CoV2-specific antibodies, other groups are studying the role of antimicrobial peptides, proteins such as lactoferrin, fatty acids, and human milk oligosaccharides (HMOs) as potential antiviral components of human milk (26–30). These results will be critical to reinforce the role of human milk in infection protection and disease prevention. This research may also guide the development of novel antiviral agents in the fight against the COVID-19 pandemic—based on the power of human milk.

## Impact of the COVID-19 Pandemic on Breastfeeding Mothers

The initial lack of knowledge and continuing misinformation around COVID-19 and human milk has impacted breastfeeding mothers around the world. First and foremost, the COVID-19 pandemic has been a cause of significant stress for breastfeeding mothers (31), with families having difficulty in accessing evidence-based lactation care and support (2). This is of importance as prenatal and postnatal anxiety have also been identified as having a negative impact on breastfeeding duration, despite breastfeeding reducing post-partum depression (5–7). Interestingly, this research includes measures of stress in the milk such as cortisol, cytokines, and serotonin as these have been shown to be related to infant temperament (32). Furthermore, as maternal nutrition may have been altered positively or negatively by the pandemic, changes in the milk microbiome may also be prevalent and thus have impact on infant development (33).

Globally, the birth and breastfeeding experiences of families have not been prioritized and care has changed in ways that may negatively impact birth outcomes and the establishment of breastfeeding. Some of these negative practices are: 1) women being forced to go through labor and birth without the presence of a partner or support person (34, 35), 2) some NICUs not allowing any parental presence at the bedside while others are limiting either the amount of time that the parents can visit or allowing only one parent to be present at a time (36), 3) some hospitals continue to separate mothers who are COVID-19 positive or a Person Under Investigation (PUI) from their infants despite WHO recommendations to avoid this practice (37), 4) discouragement of skin to skin contact and direct breastfeeding (38), 5) early discharge following birth, as early 24 h post-birth for vaginal births and 48 h post-cesarean birth (39, 40), 6) a lack of access to in person pediatric follow-up and hands-on technical breastfeeding assistance (2). Indeed, during the initial phase of the pandemic in Italy, there was concern that breastfeeding had actually been implicated as a “culprit or scapegoat,” despite available evidence suggesting that 1) few pregnant women have been severely affected by COVID-19, 2) evidence for transplacental transmission of the virus very rarely shows a clinical impact, and 3) infected neonates are generally asymptomatic, or have few symptoms, with similar infection rates with vaginal delivery or Cesarean section (41, 42).

Unfortunately, guidelines on post-partum practices for the management of SARS-CoV-2 infection, for example advice on skin to skin contact (SSC), are highly variable and hence confusing (38). Unpublished data collected by the National Center for Disease Prevention and Health Promotion, Italian National Institute of Health (ISS-Rome), from 146 COVID-19-positive women who gave birth in Italy between February and June 2020, noted that although 73% were breastfeeding (or expressing breast milk), only 15% practiced SSC, furthermore, the Italian Society of Neonatology also reported limitations of access to parents of NICU infants (43). This has implications for mothers' mental health, for even mothers of term delivered infants, these conditions adversely affected the mothers' emotional state, worsening depressive symptoms (44).

The WHO from the start of the pandemic, has consistently endorsed early exclusive breastfeeding and SSC (1, 45). If the mother is COVID-19 positive or a PUI, the mother should wear a mask, practice good hand hygiene, and avoid coughing onto her chest or even consider washing her breast with soap and water prior to feeding (46). If a mother is too unwell to direct breastfeed, the second choice is for her to express milk and have another family member feed the milk (47). If a mother is expressing milk the same guidelines should apply in terms of wearing a mask, good hand hygiene, and avoiding coughing onto her chest wall. During milk expression, the family should be provided explicit instruction for pump hygiene. The Centers for Disease Control and Prevention (CDC) offers instructions sheets in English and Spanish that can be downloaded for free from their website (48). In addition, use of a chlorine solution as an efficient decontamination medium without toxic risk has recently been described (49).

If the mother cannot breastfeed or expressed maternal milk is not available, Pasteurized Donor Human Milk (PDHM) should be considered (45). Since the virus has been shown to be destroyed by Holder Pasteurization (21, 22), PDHM would be a better choice than infant formula whenever it is available and after having prioritized the use for Very Low Birth Weight Infants (VLBWIs). We acknowledge that access to PDHM is not equal in all countries in the world. However, it is also important to note that both the European Milk Banking Association (EMBA) and the Human Milk Banking Association of North America (HMBANA) have experienced inconsistent shortages of PDHM across different states and countries (50–52). In some Italian cities such as Milan and Turin the home collection of donated human milk has been practically interrupted for 3 months during the lockdown (43). Interestingly, in some human milk bank settings, the total amount of donated milk has on the contrary increased (51), possibly due to a stronger devotion toward the expression of milk on the part of mothers during the COVID-19 pandemic, which prevailed over the logistic effect of isolation and the negative effect on the mental status of mothers.

Shenker and the Virtual Collaborative Network of Human Milk Banks report on the issues that need to be addressed to improve and secure the availability of PDHM if mother's own milk is not available (53). Global policy leaders and funding agencies must recognize and prioritize four high-impact areas: (1) ensuring neonatal nutrition (note: human milk nutrition particularly for the most vulnerable neonates) is an essential focus during emergencies; (2) research funding that improves human milk bank systems in response to new infectious threats; (3) investing in innovation across all aspects of milk banking processes to improve the responsivity, access, and quality of donor milk provision; and (4) supporting the integration of learnings and innovations by the global milk bank community during COVID-19 into newborn, nutrition, and emergency response planning for future emergencies.

## Call to Action to Protect Human Milk and Breastfeeding

Given the extensively documented benefits of human milk and breastfeeding for mothers, infants, the society, and the environment (3, 54), as well as the low likelihood of infants becoming ill from COVID-19 (41, 42), this is an opportunity for health care professionals to undertake a call to action to protect human milk and breastfeeding. Since no live virus has been detected in human milk whereas specific antibodies against SARS-CoV-2 have (18, 23, 24), a claim that cannot be attributed to infant formula, health care providers should use the current pandemic to underscore the importance of human milk and breastfeeding as a health promoting and possibly lifesaving medical intervention (55). In addition, it is of value to acknowledge that human milk and breastfeeding can play an important role in mitigating toxic stress in childhood and its effects on developmental disorders that begin early in life (56).

Globally, there are tremendous disparities in the use of human milk and breastfeeding with only 41% of infants receiving human milk for the first 6 months (57). In fact, African American families in the United States are at high risk for not making informed feeding choices and the CDC has documented that these families are also less likely to give birth in hospitals that use PDHM (58, 59).

In this regard, all families should be afforded the opportunity to make an informed feeding decision regarding the necessity of human milk and breastfeeding for their child. Research demonstrates that if families receive specific and detailed information about the science of human milk, families will make the decision to provide milk for their children (60). It is not enough to only teach the pregnant woman about breastfeeding. Her partner or support person, as well as family members should also be taught the science of human milk and the physiology of lactation. In order to achieve this goal, the current practice paradigm for prenatal care must be dramatically changed (61). Families need to be educated on the science of human milk and the physiology of lactation. Health care providers must educate families that milk production begins at 16 weeks of gestation, so as long as breasts are present, milk is being produced. Health care providers must conduct thorough prenatal lactation risk assessment and should help families understand risk factors that may influence development of milk supply (62). In addition, families who have risk factors such as breast surgery or abnormal breast tissue development should receive appropriate guidance to facilitate achievement of maximal milk supply after birth (63). Health care professionals should provide parents with appropriate prenatal anticipatory guidance and support a pro-active management strategy after birth to ensure that a copious milk supply in achieved (61). Group prenatal care (centering model) by midwives as well as the use of doulas in breastfeeding guidance pre- and post-birth would be powerful interventions to improve breastfeeding outcomes (60, 64). Early SSC and direct breastfeeding should always be the priority. With the staff reductions in maternity services, the lack of access to lactation professionals, and early hospital discharge, families need to receive appropriate anticipatory prenatal guidance. Moreover, maternity hospital practices facilitating breastfeeding, such as rooming-in, should be implemented overcoming the well-known constraints and following the narrative suggestions from mothers (65).

It is also of paramount importance for policy makers to protect human milk and breastfeeding during this pandemic and beyond. One such example is the work of the United States Breastfeeding Committee COVID-19 Infant and Young Child Feeding Constellation (66). This group has been meeting since early in the pandemic collaborating with leading public health partners working at every level of the system with experience in responding to public health emergencies (66). As noted in this manuscript, despite WHO recommendations to promote and protect human milk and breastfeeding, this has not occurred at the local level. Therefore, it is imperative that policy makers in all countries and at all system levels prioritize the protection of human milk and breastfeeding, and of PDHM as a bridge where warranted.

## Conclusions

In conclusion, the current pandemic has uncovered the urgent and immediate need to invest in research that establishes the safety of human milk and safety at crisis onset. Otherwise, the gap in knowledge leads to fear-based confusion, misinformation, and the amplified risk of breastfeeding cessation despite the well-documented benefits of human milk and breastfeeding. Following the immediate and rigorous safety evaluation performed by a mobilized human milk research community, human milk has been shown to provide an immense opportunity to help combat the current COVID-19 pandemic threat. It is of paramount importance that we continue to use the growing evidence base in order to prioritize the use of human milk and breastfeeding. Utilization of the models that focus on informed decision making, initiation and maintenance of milk supply, SSC, and direct breastfeeding have demonstrated significant increases in human milk rates at discharge and beyond (67–69). We need to improve equal access for all families to ensure the maintenance and growth of evidence-based lactation education and care during the course of the COVID-19 pandemic and indeed, beyond.

## Author Contributions

All authors listed have made a substantial, direct and intellectual contribution to the work, and approved it for publication.

## Conflict of Interest

JG serves as member of the National Health Council and is also director of the Dutch national Human Milk Bank. The remaining authors declare that the research was conducted in the absence of any commercial or financial relationships that could be construed as a potential conflict of interest.
